# Salvianolic Acid B Prevents Arsenic Trioxide-Induced Cardiotoxicity *In Vivo* and Enhances Its Anticancer Activity *In Vitro*

**DOI:** 10.1155/2013/759483

**Published:** 2013-04-08

**Authors:** Min Wang, Guibo Sun, Ping Wu, Rongchang Chen, Fan Yao, Meng Qin, Yun Luo, Hong Sun, Qiang Zhang, Xi Dong, Xiaobo Sun

**Affiliations:** ^1^Institute of Medicinal Plant Development, Chinese Academy of Medical Sciences & Peking Union Medical College, Beijing 100193, China; ^2^Guang'anmen Hospital, China Academy of Chinese Medical Sciences, Beijing 100053, China; ^3^School of Biological Sciences, University of Edinburgh, King's Buildings, Edinburgh EH9 3JU, UK; ^4^Academy of Chinese Materia Medica, Wenzhou Medical College, Wenzhou 325035, China

## Abstract

Clinical attempts to reduce the cardiotoxicity of arsenic trioxide (ATO) without compromising its anticancer activities remain to be an unresolved issue. In this study, we determined whether Sal B can protect against ATO-induced cardiac toxicity *in vivo* and increase the toxicity of ATO toward cancer cells. Combination treatment of Sal B and ATO was investigated using BALB/c mice and human hepatoma (HepG2) cells and human cervical cancer (HeLa) cells. The results showed that the combination treatment significantly improved the ATO-induced loss of cardiac function, attenuated damage of cardiomyocytic structure, and suppressed the ATO-induced release of cardiac enzymes into serum in BALB/c mouse models. The expression levels of Bcl-2 and p-Akt in the mice treated with ATO alone were reduced, whereas those in the mice given the combination treatment were similar to those in the control mice. Moreover, the combination treatment significantly enhanced the ATO-induced cytotoxicity and apoptosis of HepG2 cells and HeLa cells. Increases in apoptotic marker cleaved poly (ADP-ribose) polymerase and decreases in procaspase-3 expressions were observed through western blot. Taken together, these observations indicate that the combination treatment of Sal B and ATO is potentially applicable for treating cancer with reduced cardiotoxic side effects.

## 1. Introduction

Arsenic trioxide (ATO) was discovered more than 2000 years ago for treating diseases such as cancer, syphilis, and tuberculosis [1]. Recently, ATO has been recognized as a clinically effective drug for treating acute promyelocytic leukemia (APL) [2]. However, this treatment was also associated with QT prolongation, torsades de pointes, and sudden death [3–5], thereby restricting its broad application. The possible mechanisms of ATO-induced cardiotoxicity include DNA fragmentation, reactive oxygen species (ROS) generation, cardiac ion channel changes, and apoptosis [6–8]. Although the exact mechanism of ATO cardiotoxicity is not currently known, the generation of ROS is very common in arsenic toxicity [8, 9]. Therefore, antioxidative agents could provide an alternative approach to treat ATO-induced cardiac damages.

Salvianolic acid B (Sal B), the most abundant and bioactive member of the salvianolic acids in *Salvia miltiorrhiza* [10], has strong antioxidant and free radical scavenging activities. It is a major component of the commercial Fufang Danshen products, such as the Compound Danshen Dripping Pill, the Danshen Pian, and the Danshen Injection, used for the treatment of coronary heart disease, angina pectoris, and other heart diseases [11, 12]. The cardioprotective properties of Sal B are partly attributed to its antioxidant and antiapoptotic effects [13, 14]. We previously demonstrated that Sal B can ameliorate ATO-induced cardiac cell injury * in vitro* by preventing ATO-induced excessive intracellular ROS and cardiomyocyte apoptosis [15]. This finding has prompted us to investigate whether Sal B can protect against ATO-induced adverse effects *in vivo* without changing the anticancer activity of ATO.

In this work, we examined the effect of an ATO and Sal B combination treatment on the development of cardiotoxic side effects *in vivo* using a mouse model. We then examined the combination treatment * in vitro* on human hepatoma (HepG2) cells and human cervical cancer (HeLa) cells. The results demonstrated that the combination treatment eliminated the cardiotoxic side-effects of ATO and enhanced ATO anticancer activities against HepG2 and HeLa cells * in vitro*.

## 2. Materials and Methods

### 2.1. Chemicals and Materials

Sal B (>98% purity) was purchased from Shanghai Winherb Medical S & T Development Co., Ltd., (Shanghai, China). ATO was purchased from Harbin Yi-da Pharmaceutical Ltd., (Harbin, China). HepG2 and HeLa cells were obtained from the Cell Bank of the Chinese Academy of Sciences (Shanghai, China). Dulbecco's modified Eagle's medium (DMEM) and fetal bovine serum (FBS) were purchased from Gibco BRL (Grand Island, NY, USA). 3-(4,5-dimethylthiazol-2-yl)-2,5-diphenyltetrazolium bromide (MTT) was purchased from Sigma-Aldrich (St. Louis, MO, USA). Annexin V/propidium iodide (PI) apoptosis detection kit was obtained from Invitrogen (Eugene, USA). Kits for determining total creatine kinase (CK), aspartate aminotransferase (AST), lactate dehydrogenase (LDH), catalase (CAT) activity, glutathione peroxidase (GSH-Px) activity, and superoxide dismutase (SOD) activity were obtained from Jiancheng Bioengineering Institute (Nanjing, China). The primary antibodies against poly (ADP-ribose) polymerase (PARP), procaspase-3, Bcl-2, Bax, p-Akt, and Akt were purchased from Santa Cruz Biotechnology (Santa Cruz, CA, USA). The *β*-actin primary antibodies and horseradish-peroxidase-(HRP-)conjugated secondary antibodies were purchased from CWBiotech (Beijing, China). All chemical reagents were of analytical grade and commercially available.

### 2.2. Animals

Male BALB/c mice weighing from 18 g to 20 g were purchased from Beijing Vital River Laboratory Animal Technology Co., Ltd., Beijing, China. The animals were housed under standard laboratory conditions (25°C ± 1°C temperature, 60% humidity, and 12 h photoperiod) and given free access to sterile food and water. All procedures were performed in accordance with the China Physiological Society “Guiding Principles in the Care and Use of Animals” and with the approval of the Laboratory Animal ethics Committee of Institute of Medicinal Plant Development, Peking Union Medical College.

### 2.3. Experimental Protocols

A total of 60 mice were randomly assigned to four groups: control (10 mL/kg saline), ATO-treated (1 mg/kg ATO), ATO and Sal B combination-treated (2 mg/kg Sal B 1 h before ATO administration), and Sal B treated (2 mg/kg Sal B). All treatments were injected via the tail vein for 2 weeks.

### 2.4. Echocardiography for Cardiac Functional Analysis

Cardiac function was analyzed using echocardiography 24 h after drug treatment. The mice were anesthetized using from 1.5% to 2% isoflurane, and M-mode ultrasound images were obtained using a Vevo 770 micro-ultrasound system (VisualSonics, Toronto, ON, Canada).

### 2.5. Plasma Collection and Biochemical Determination

After the completion of the echocardiography, blood samples were obtained from the inner canthus using a capillary tube under chloral hydrate anesthesia. The samples were centrifuged at 3000 ×g for 15 min within 1 h after collection. The activities of LDH, CK, AST, GSH-PX, CAT, and SOD in the plasma were determined using commercially available kits purchased from Jiancheng Bioengineering Institute (Nanjing, China) according to the manufacturer's instructions.

### 2.6. Histopathology

The apex of the heart was fixed in 10% formalin, routinely processed, and then embedded in paraffin. Paraffin sections (3 mm) were placed on glass slides, stained with hematoxylin and eosin (HE), and then examined under a light microscope (CKX41, Olympus, Tokyo, Japan) by a pathologist blinded to the groups studied.

### 2.7. Cell Culture and Treatment

HepG2 and HeLa cells were obtained from the Cell Bank of the Chinese Academy of Sciences (Shanghai, China). The cells were cultured in DMEM supplemented with 10% (v/v) FBS, 2 mM L-glutamine, 100 U/mL penicillin, and 100 *μ*g/mL streptomycin and maintained in a humidified incubator with 95% air and 5% CO_2_ at 37°C. The cells were subcultured after reaching from 70% to 80% of confluence. In all experiments, the cells were plated at an appropriate density according to the experimental design and were grown for 36 h before experimentation. The optimal doses of Sal B and Sal B and ATO combination treatment were determined in our preliminary study (see Supplementary Data 4). Four sets of experiments were performed: (1) control cells; (2) cells treated with ATO (4 *μ*M) for 48 h; (3) cells treated with Sal B alone (10 *μ*M) for 48 h; and (4) cells cotreated with 4 *μ*M ATO and 10 *μ*M Sal B for 48 h.

### 2.8. Cell Viability Analysis

Cell viability was determined using MTT assay. Briefly, HepG2 or HeLa cells were seeded on 96-well plates at a density of 5 × 10^3^ cells/well. The cells were treated for 48 h with ATO alone (4 *μ*M), Sal B alone (10 *μ*M), or in combination (4 *μ*M ATO and 10 *μ*M Sal B). Subsequently, the wells added with 20 *μ*L MTT (5 mg/mL) were incubated for 4 h. The medium was removed, and the formazan crystals were dissolved in DMSO. Absorbance was read at 570 nm on a microplate reader (MQX 200, BioTek Instruments, Winooski, VT, USA).

### 2.9. Annexin V-FITC/PI Apoptosis Assay

The percentage of early apoptosis and necrosis was measured using an Annexin V-FITC/PI apoptosis kit for flow cytometry according to the manufacturer's instructions (Invitrogen). After treatment, the cells were harvested, washed twice with cold phosphate-buffered saline (PBS), and then incubated with 5 *μ*L FITC-Annexin V and 1 *μ*L PI working solution (100 *μ*g/mL) for 15 min in the dark at room temperature. Cellular fluorescence was measured by flow cytometry analysis using a flowcytometer (FACS Calibur, BD Biosciences, CA, USA).

### 2.10. Western Blot Analysis

After the designated treatment, heart tissue or HepG2 and HeLa cells were lysed for 30 min on ice with T-PER Tissue or Cell Protein Extraction Reagent (Pierce Chemical Co., Rockford, IL, USA) containing 1% phenylmethylsulfonylfluoride. A clear lysate was obtained through centrifugation at 12000 ×g for 15 min at 4°C. The supernate was collected. Protein concentration was determined through bicinchoninic acid assay. Equal amounts of lysates (10 *μ*g) were fractionated using 10% sodium dodecyl sulfate polyacrylamide gel and electrotransferred onto nitrocellulose membranes. The membranes were blocked for 1 h in 5% skim milk and incubated overnight at 4°C with the primary antibodies. The membranes were washed for 30 min and then incubated with HRP-conjugated secondary antibodies for 1 h at room temperature. The membranes were washed for 30 min and then visualized using enhanced chemiluminescence. The protein expression levels were determined by analyzing the signals captured on the nitrocellulose membranes using a ChemiDoc image analyzer (Bio-Rad, USA). 

### 2.11. Statistical Analysis

Data from at least three independent experiments were expressed as means ± SD. Statistical comparisons between different groups were measured using one-way ANOVA followed by the Student-Newman-Keuls test. The level of significance was set at *P* < 0.05.

## 3. Results

### 3.1. Combination Treatment with Sal B and ATO Decreases the Incidence of Cardiotoxic Side Effects Compared with ATO Treatment Alone in BALB/c Mouse Models

#### 3.1.1. Sal B Attenuates ATO-Induced Cardiac Dysfunction

**Figure 1:**
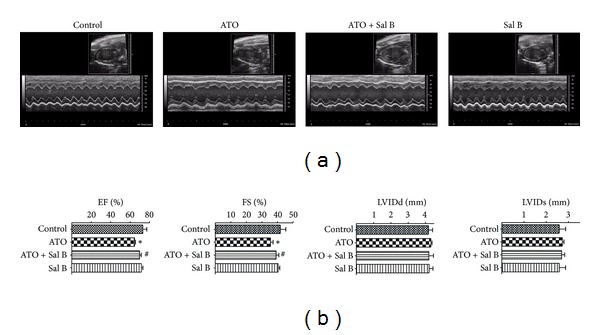
Effects of Sal B and ATO on cardiac dysfunction. Mice were treated with saline or ATO with or without Sal B pretreatment. Echocardiography was performed after 2 weeks. (a) Representative M-mode echocardiograms were shown. (b) Changes in ejection fraction (EF), fractional shortening (FS), left ventricular internal diameter at diastole (LVIDd), and left ventricular internal diameter at systole (LVIDs) in the four groups. Values (*n* = 15 per group) are expressed as means ± SD. *P < 0.05 versus control; ^#^ *P* < 0.05 versus ATO-treated mice.

We examined the effect of the combination treatment using an *in vivo* mouse model of ATO-induced cardiotoxicity. The mice were treated with ATO and Sal B alone or a combination of both for 2 weeks, and the results were examined using M-mode echocardiography (Figure 1(a)). Compared with the control group, both ejection fraction and fractional shortening significantly decreased in the mice administered with ATO treatment alone but significantly increased in the mice given the combination treatment (Figure 1(b)). However, no significant differences were observed in the left ventricular dimensions at the systole and diastole between the groups. 

#### 3.1.2. Sal B Prevents Myocardial Damage

**Figure 2:**
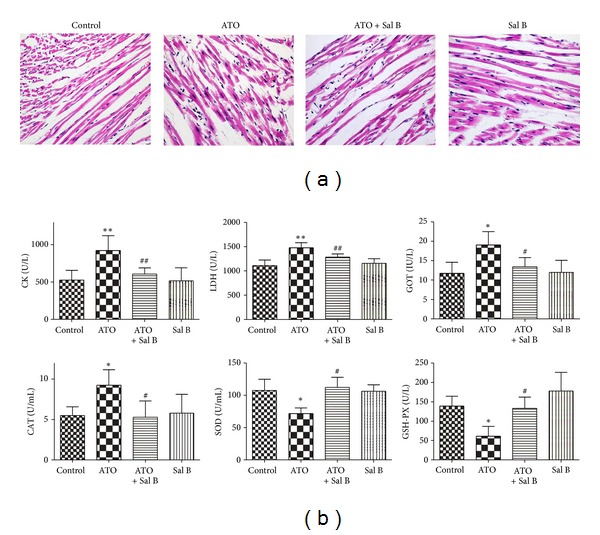
Effects of Sal B on ATO-induced myocardial injury *in vivo*. (a) Effects of Sal B on histological changes in mice hearts by HE (hematoxylin and eosin) staining (×200). (b) Effects of Sal B on the activities of CK, LDH, GOT, CAT, SOD, and GSH-PX in plasma. Values (*n* = 15 per group) are expressed as means ± SD. *P < 0.05 versus control; **P < 0.01 versus control; ^#^ *P* < 0.05 versus ATO-treated mice; ^##^ *P* < 0.01 versus ATO-treated mice. CK: creatine kinase; LDH: lactate dehydrogenase; GOT: glutamic oxaloacetic transaminase; CAT: catalase; SOD: superoxide dismutase; GSH-PX: glutathione peroxidase.

An overall view of the distribution of myocardial damage at the light microscopy level is shown in Figure 2(a). HE staining of the cardiac tissues showed clear structural abnormalities, including cytoplasmic vacuolization, myofibrillar loss, and cardiomyocyte necrosis, in the ATO-treated hearts compared with the control group. Structural abnormalities in the ATO-treated hearts were partly prevented by the combination-treated group. The Sal B-treated group had normal myocardial morphology.

Next, we measured the levels of serum cardiac enzymes (CK, AST, and LDH), which are the biomarkers used for monitoring myocardial damage [16]. Combination treatment significantly attenuated the ATO-induced increase in serum cardiac enzyme levels. Sal B treatment alone did not show any obvious abnormalities compared with the control treatment (Figure 2(b)).

#### 3.1.3. Sal B Enhances Antioxidant Capacity

As shown in Figure 2(b), the activities of GSH-PX and SOD in the ATO-treated group were reduced compared with those in the control group. However, GSH-PX and SOD activities in the combination-treated group increased compared with those in the ATO-treated group. In addition, plasma CAT activity in the ATO-treated group decreased compared with that in the control group. This reduction was reversed by the combination treatment. These findings suggest that Sal B could considerably improve cellular antioxidative defense capacity against ATO-induced oxidative stress.

#### 3.1.4. Sal B Increases the Expression of Prosurvival Proteins in Heart Tissue

**Figure 3:**
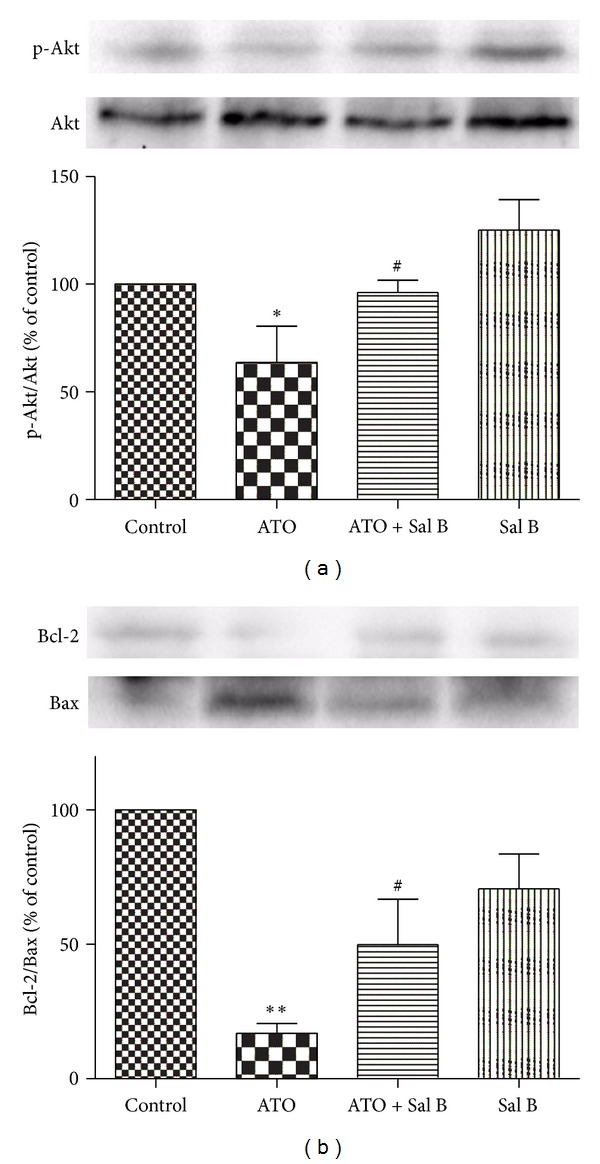
Effects of Sal B treatment on protective protein expressions in heart tissue *in vivo*. Western blotting was performed on harvested ventricular tissue from mice after 2 weeks of treatment with ATO, Sal B, or both. (a) Effects of Sal B on Akt phosphorylation. Quantitative analysis of the ratio of p-Akt to Akt in protein expression was evaluated. (b) Effects of Sal B on Bcl-2 and Bax. Quantitative analysis of the ratio of Bcl-2 to Bax in protein expression was evaluated. Data are representative of three different experiments. *P < 0.05 versus control; **P < 0.01 versus control; ^#^ *P* < 0.05 versus ATO-treated mice; ^##^ *P* < 0.01 versus ATO-treated mice.

The levels of pAkt and Bcl-2 decreased in the ATO-treated group but not in the combination-treated group (Figures 3(a) and 3(b)). By contrast, the Bax level increased in the ATO-treated group. This increase was not observed in the combination-treated group, in which the Bax level was similar to that in the control group. Our results indicate that the Sal-B-induced upregulation of Akt and Bcl-2 proteins may be involved in the cardioprotective effects of the combination treatment. These results confirm that the combination treatment can promote the expressions of prosurvival proteins in the heart.

### 3.2. Combination Treatment Reduces Cell Viability in HepG2 and HeLa Cancer Cell Lines

**Figure 4:**
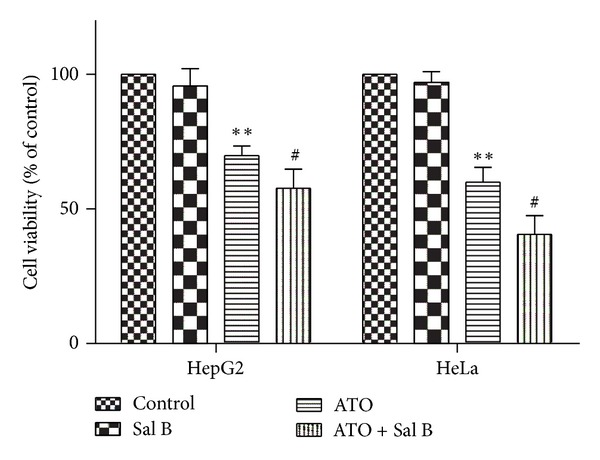
Effects of Sal B and ATO treatment on the cell viability of cancer cells. HepG2 and HeLa cells were treated with ATO (4 *μ*M) in the presence or absence with Sal B (10 *μ*M) for 48 h, respectively. Cell viability was determined by MTT assay (expressed as the percentage of control in each cell line). The data are expressed as means ±** **SD from three independent experiments. **P < 0.01 versus control; ^#^ *P* < 0.05 versus ATO-treated cells; ^##^ *P* < 0.01 versus ATO-treated cells.

Through MTT assay, we examined the viability of HepG2 and HeLa cell lines after treatment with ATO and Sal B alone (4 and 10 *μ*M, resp.) or a combination of both. As shown in Figure 4, combination-treated cells had a significantly lower viability than those cells treated with ATO alone. In the normal H9c2 cell lines, combination-treated cells had a higher viability than those cells treated with ATO alone (see Supplementary Data 3). This finding indicates that combination treatment may have the potential to enhance the antitumor effect of ATO. 

### 3.3. Combination Treatment Induces Apoptosis in HepG2 Cells and HeLa Cells

**Figure 5:**
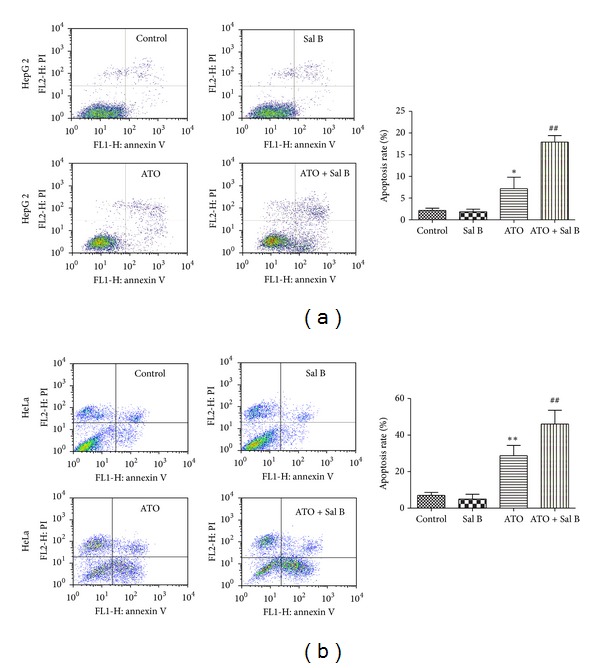
Effects of Sal B on the apoptosis of cancer cells induced by ATO. Annexin V-FITC/PI flow-cytometric data from HepG2 (a) and HeLa (b) cells treated with ATO, Sal B, or both (4 *μ*M ATO, 10 *μ*M Sal B) for 48 h. The data are expressed as means ± SD from three independent experiments. **P < 0.01 versus control; ^#^ *P* < 0.05 versus ATO-treated cells; ^##^ *P* < 0.01 versus ATO-treated cells.

To analyze the effect of Sal B and ATO combination treatment on apoptosis, we performed annexin V/PI flow cytometry. The apoptotic fraction in the ATO and Sal B combination-treated group significantly increased compared with that in the ATO-group or Sal B-treated group in HepG2 and HeLa cells (Figures 5(a) and 5(b)). The results indicate that the combination treatment can cause considerably higher apoptosis in cancer cells than ATO or Sal B alone at the tested concentrations.

### 3.4. Combination-Treatment-Induced Apoptosis Is Caspase Dependent in HepG2 Cells and HeLa Cells

**Figure 6:**
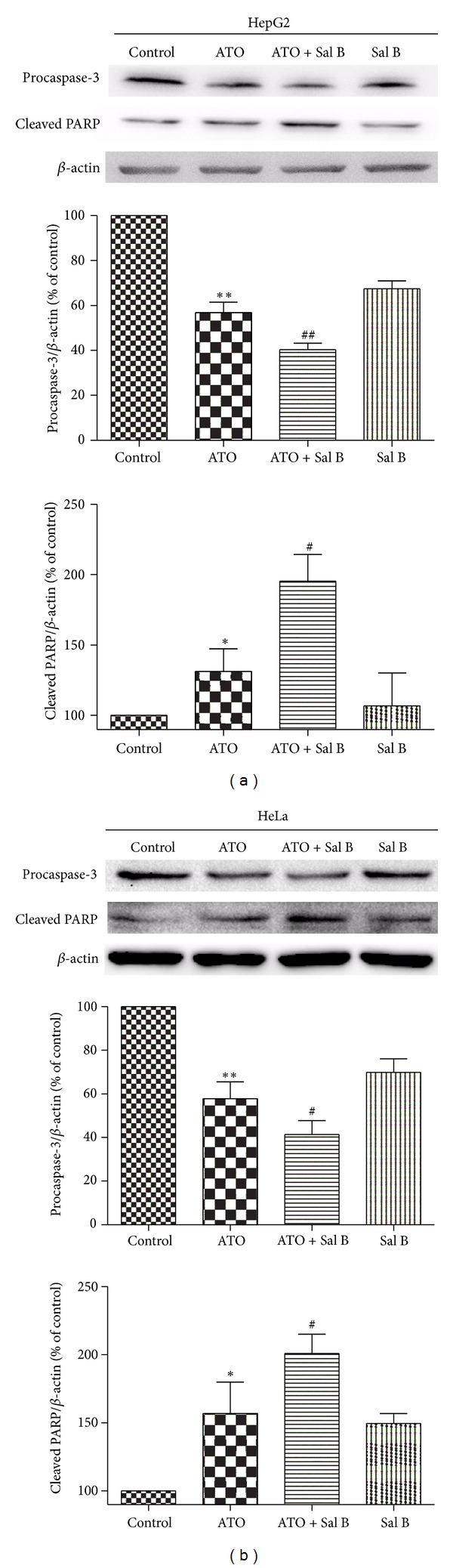
Effects of Sal B and ATO treatment on apoptosis-related proteins * in vitro*. Western blotting was performed on HepG2 (a) and HeLa (b) cells treated with ATO, Sal B, or both (4 *μ*M ATO, 10 *μ*M Sal B) for 48 h. The activation of caspase-3 is reflected by the reduced amount of procaspase-3, and the activation of PARP is reflected by the amount of the cleaved PARP. *β*-actin served as an internal control. The data are expressed as means ± SD from three independent experiments. *P < 0.05 versus control; **P < 0.01 versus control; ^#^ *P* < 0.05 versus ATO-treated cells; ^##^ *P* < 0.01 versus ATO-treated cells.

We tested the involvement of caspase-3, the major effector of caspase, and PARP, the main substrate of caspases. Activation of caspase-3 was assessed by the decreased amount of procaspase-3 based on the western blot analysis [17]. As shown in Figures 6(a) and 6(b), the levels of procaspase-3 in the ATO and Sal B combination-treated group significantly decreased compared to that in the ATO-treated group in both HepG2 cells and HeLa cells, indicating the enhanced cleavage of procaspase-3 to its active form. Correspondingly, PARP cleavage was the most pronounced in the ATO and Sal B combination-treated group (Figures 6(a) and 6(b)). The results show that the combination treatment can induce greater acceleration of apoptosis in cancer cells than ATO or Sal B alone at the tested concentrations.

## 4. Discussion

ATO attracted worldwide attention in the past decade because of its remarkable efficacy in acute APL [18]. However, the therapeutic use of ATO is limited by its cardiac toxicity. Therefore, the development of strategies to reduce these cardiotoxic effects would allow us to explore the full therapeutic potential of ATO, with a substantial impact on cancer therapy. This study, combined with our previous report [15], provides evidence that Sal B can protect against ATO-induced cardiotoxicity and enhance its anticancer activity.

Many reports revealed that Sal B is a promising compound for cardiovascular therapy and may act on multiple targets, leading to a concerted positive effect [13]. In the present study, we showed that Sal B significantly prevented ATO-induced cardiotoxicity. In agreement with several other studies [7, 19, 20], the underlying mechanism is partly associated with its cellular antioxidative defense capacity against ATO-induced oxidative stress, which is a significant increase in the activities of GSH-PX and SOD in plasma. These results are also further confirmed by western blot (see Supplementary Data 1). In addition to oxidative damage, our results showed that ATO also caused changes in cardiac function and increased the levels of serum cardiac enzymes, whereas Sal B treatment was found to be effective in preventing ATO-induced alteration in the heart. Histological studies confirmed that ATO treatment caused clear physiological changes in the cardiac tissue. However, Sal B treatment could prevent this change and could also maintain the cardiac tissue physiologically similar to that of the normal control. Furthermore, the protective effect of the combination treatment was also found in the expression of proteins in the heart. Consistent with the * in vitro* data [15], both p-Akt and Bcl-2 levels were significantly reduced in the ATO-treated group. However, the expression levels of both proteins were recovered in the combination-treated group. In cancer cells, however, the effects of the combination are precisely the opposite (see Supplementary Data 2). These results suggest that Sal B possesses antioxidant activities and cardioprotective effects against ATO-induced myocardial damages.

Another equally important issue is if Sal B could decrease ATO anticancer activity. Recent studies have demonstrated that Sal B exhibited significant growth arrest and apoptosis in many human cancer cell lines, such as head and neck as well as liver and HL-60, with no apparent toxicity to noncancerous cells [21, 22]. Notably, reports showed that Sal B could inhibit CYP1A2 and CYP3A4 mRNA expression and induce GST protein expression, indicating that Sal B may be a protective compound against cancer [23]. In the current study, we demonstrated that Sal B did not restrain the antiproliferative effects of ATO in HepG2 and HeLa cancer cells but enhanced its antitumor activity. This result is different from its effects on H9C2 cells. A similar trend was observed in the percentage of apoptotic cells in the combination-treated group. We further examined the involvement of caspases in cotreatment-mediated apoptosis. Compared with the control group, the expression of procaspase-3 decreased after co-treatment for 48 h. PARP is the substrate for effector caspases during apoptosis [24]. PARP cleavage is the hallmark of apoptosis [24, 25]. Caspase-mediated PARP cleavage showed that Sal B could increase the sensitivity of HepG2 cells and HeLa cells toward ATO via caspase pathways. All these results suggest that Sal B has the potential to be an adjuvant in future clinical application. 

ATO induced apoptosis in both cancer cells and cardiomyocytes, but Sal B and ATO combination could provide both cytotoxicity and cardioprotective properties, indicating that the different effects of Sal B and ATO are dependent on cell type. These results were also observed in several other antioxidant studies [7, 26]. Although the mechanism is still elusive, we speculate that it may be due to the functionally contradictory effects of some proteins critical for apoptotic signaling in different cell types induced by ATO, such as the proapoptotic effects of c-Jun-NH2-kinase (JNK) activation in ATO-induced ovarian cancer cells [27] in contrast to the antiapoptotic effects on JNK activation in some other cell lines [28, 29]. Further studies will be conducted to determine this mechanism.

In conclusion, we confirmed the protective effects of Sal B against ATO-induced cardiotoxicity *in vivo* mouse models in terms of cardiac function, morphological changes, and antioxidant enzyme activities. A combination treatment of ATO and Sal B can allow for greater toxicity to cancer cells. These data support the cardioprotective properties of Sal B against ATO-induced myocardial toxicity as well as the enhancement of anticancer activities of ATO, indicating that the combination of Sal B with ATO has potential clinical application.

## Supplementary Material

The optimal doses of Sal B and Sal B and ATO combination treatment were determined in our preliminary study (see Supplementary Data 4). In the normal H9c2 cell lines, combination-treated cells had a higher viability than those cells treated with ATO alone (see Supplementary Data 3). These results are also further confirmed by western blot (see Supplementary Data 1). In cancer cells, however, the effects of the combination are precisely the opposite (see Supplementary Data 2).
