# Supplementary material for: Salvianolic Acid B Prevents Arsenic Trioxide-Induced Cardiotoxicity In Vivo and Enhances Its Anticancer Activity In Vitro
Source: Evid Based Complement Alternat Med. 2013 Apr 8;2013:759483. doi: 10.1155/2013/759483 (PMC3638618; doi:10.1155/2013/759483)

Supplementary Data

Supplementary Data 1 Effects of Sal B treatment on SOD and GPx expressions in heart tissue in vivo. Western blotting was performed on harvested ventricular tissue from mice after 2 weeks of treatment with ATO, Sal B, or both. Data are representative of three different experiments. **P < 0.01 versus control; #p<0.05 versus ATO-treated mice.

Supplementary Data 2 Effects of Sal B and ATO treatment on Akt and Bcl-2/Bax protein expression levels in vitro. Western blotting was performed on HepG 2 (A) and HeLa (B) cells treated with ATO, Sal B, or both (4 μM ATO, 10 μM Sal B) for 48 h. Quantitative analyses of the ratio of p-Akt to Akt and the ratio of Bcl-2 to Bax in protein expression were evaluated, respectively. The data are expressed as means ± SD from three independent experiments. *p<0.05 versus control; **P < 0.01 versus control; #P < 0.05 versus ATO-treated cells.

Supplementary Data 3 Effects of Sal B and ATO treatment on the cell viability of normal H9c2 cardiomyoblast cells. H9c2 cells were treated with ATO (4 μM) in presence or absence with Sal B (10 μM) for 48 h, respectively. Cell viability was determined by MTT assay (expressed as the percentage of control in each cell line). The data are expressed as means ± SD from three independent experiments. **P < 0.01 versus control; ##P < 0.01 versus ATO-treated cells.

Supplementary Data 4 Effects of Sal B and Sal B and ATO combination treatment on the cell viability of cancer cells. HepG 2 (A) and HeLa (B) cells were incubated with increasing concentrations of Sal B (0 μM to 100 μM) for 48 h. HepG 2 (C) and HeLa (D) cells were treated with different concentrations of ATO (0-8 μM) in presence or absence with Sal B (10 μM) for 48 h, respectively. Cell viability was determined by MTT assay (expressed as the percentage of control in each cell line). The data are expressed as means ± SD from three independent experiments. **P < 0.01 versus control; #P < 0.05 versus ATO-treated cells.

Supplementary Data 1

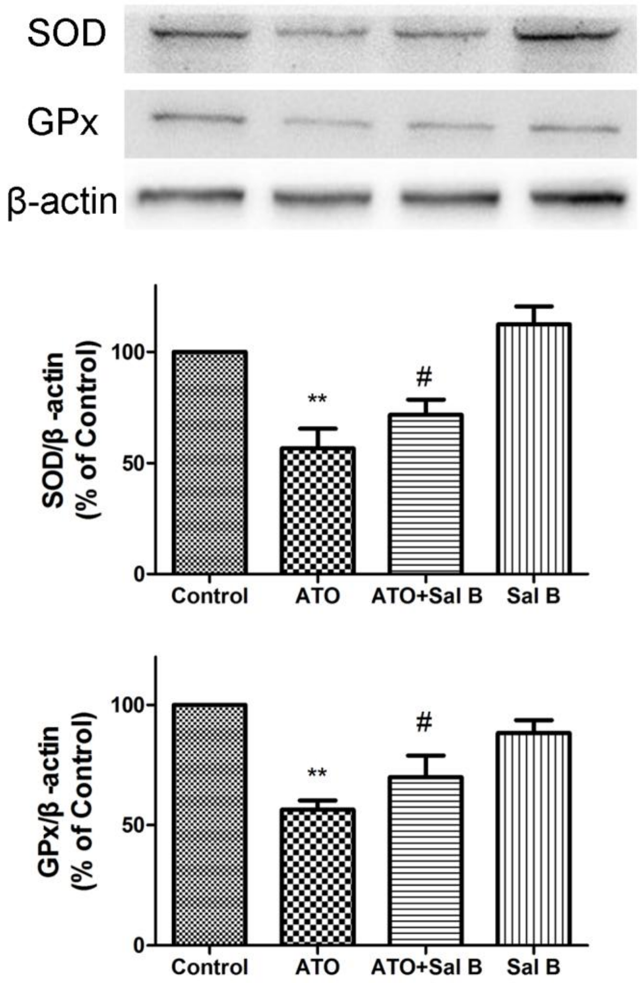

Supplementary Data 2

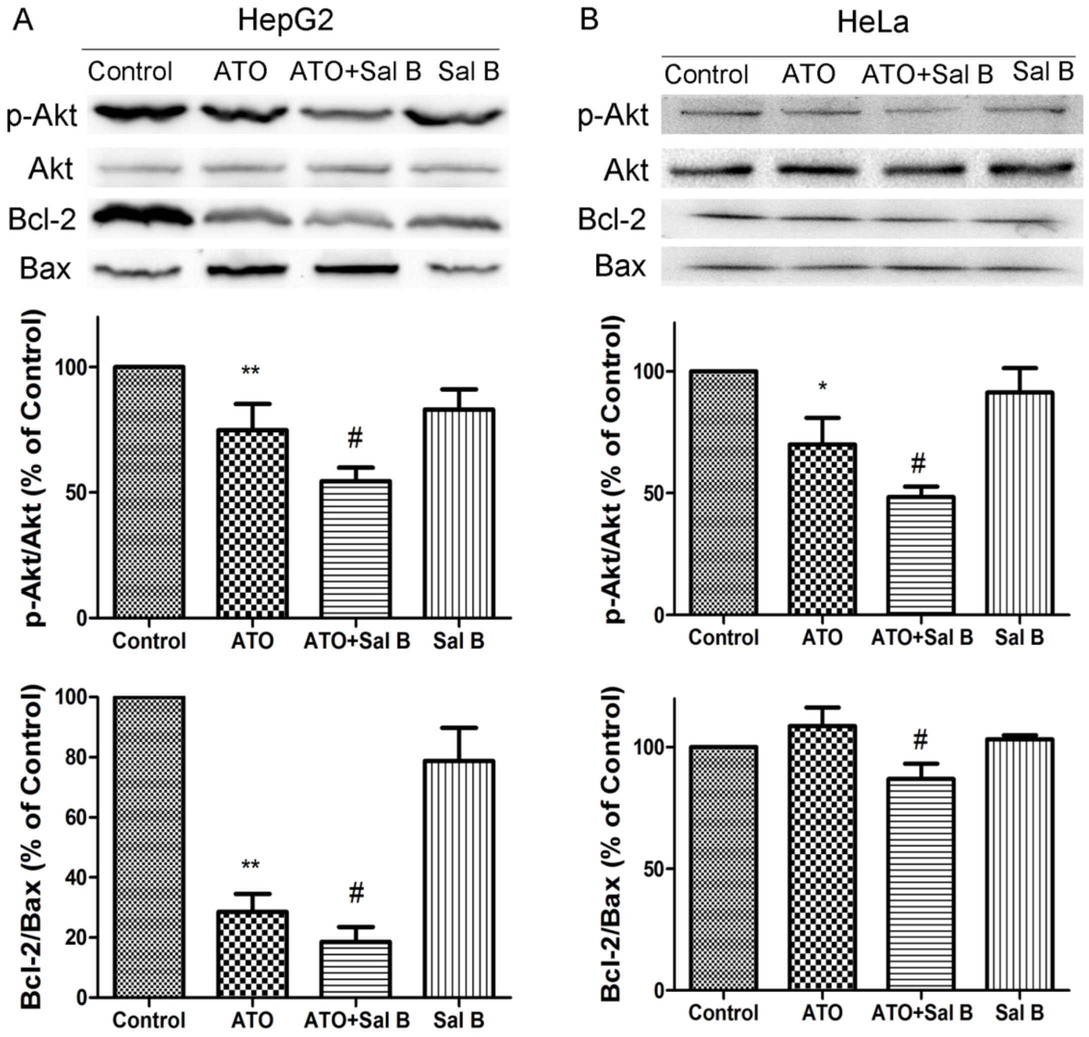

Supplementary Data 3

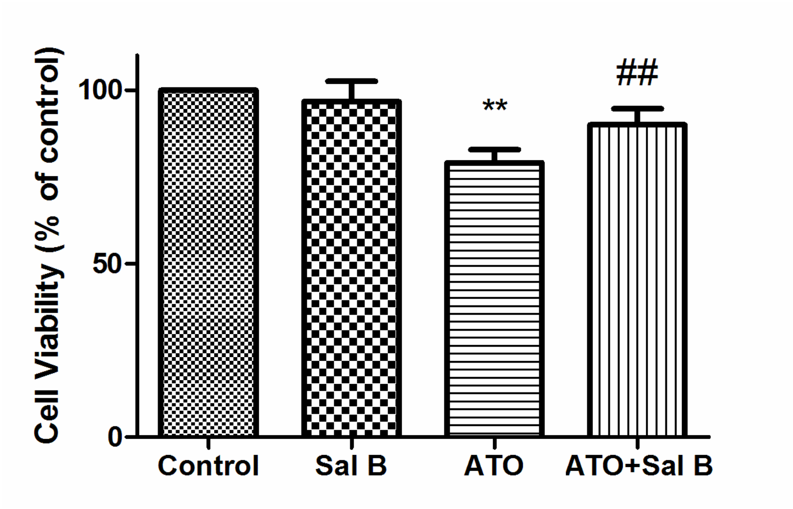

Supplementary Data 4

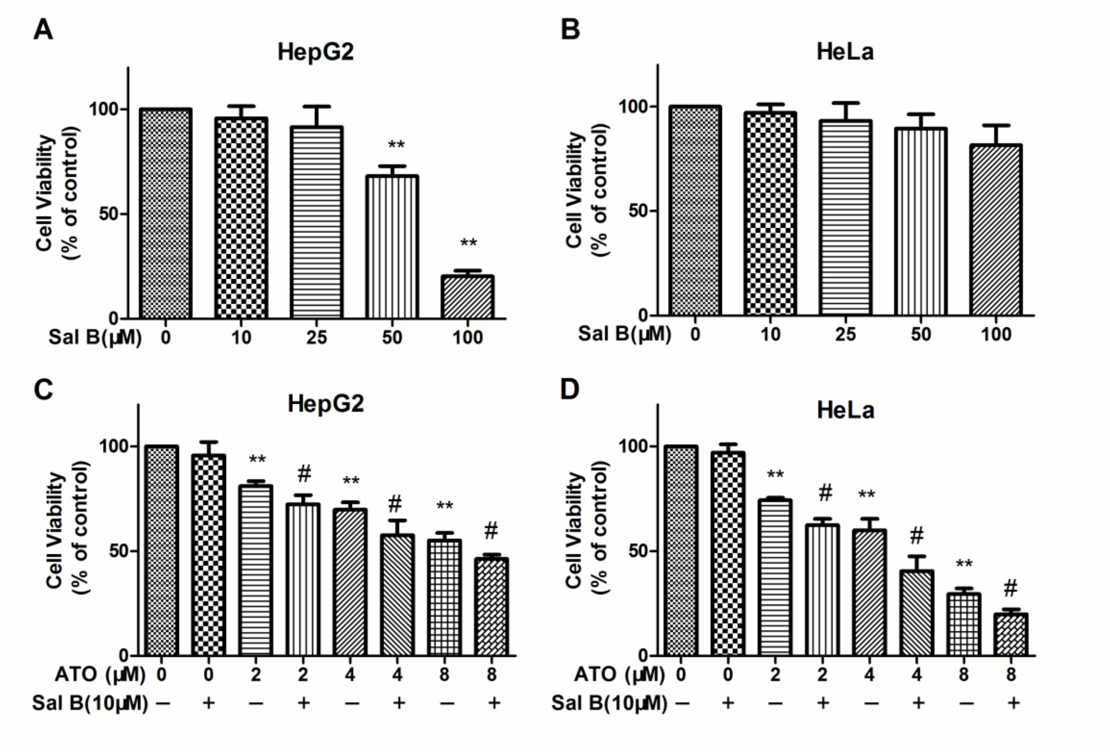

Supplement: Supplementary file 1 — The optimal doses of Sal B and Sal B and ATO combination treatment were determined in our preliminary study (see Supplementary Data 4). In the normal H9c2 cell lines, combination-treated cells had a higher viability than those cells treated with ATO alone (see Supplementary Data 3). These results are also further confirmed by western blot (see Supplementary Data 1). In cancer cells, however, the effects of the combination are precisely the opposite (see Supplementary Data 2). [file 759483.f1.pdf]
